# Morphological Comparison of Residual Ridge in Impression for Removable Partial Denture between Digital and Conventional Techniques: A Preliminary In-Vivo Study

**DOI:** 10.3390/jcm12227103

**Published:** 2023-11-15

**Authors:** Yurika Ishioka, Junichiro Wada, Eung-Yeol Kim, Kazuki Sakamoto, Yuki Arai, Natsuko Murakami, Toshiki Yamazaki, Kensuke Takakusaki, Hironari Hayama, Miona Utsumi, Shusuke Inukai, Noriyuki Wakabayashi

**Affiliations:** 1 Department of Advanced Prosthodontics, Tokyo Medical and Dental University—TMDU, 1-5-45, Yushima, Bunkyo-ku, Tokyo 113-8510, Japan; y.ishioka.rpro@tmd.ac.jp (Y.I.); kim.ey.rpro@gmail.com (E.-Y.K.); n.murakami.rpro@tmd.ac.jp (N.M.); t.yamazaki.rpro@tmd.ac.jp (T.Y.); k.takakusaki.rpro@tmd.ac.jp (K.T.); h.hayama.rpro@tmd.ac.jp (H.H.); utsumi.rpro@tmd.ac.jp (M.U.); shu.inukai.rpro@tmd.ac.jp (S.I.); wakabayashi.rpro@tmd.ac.jp (N.W.); 2 Department of Biomaterials Science, Turku Clinical Biomaterials Centre—TCBC, Institute of Dentistry, University of Turku, Itäinen Pitkäkatu 4B, 20520 Turku, Finland; 3 Department of Orthodontics, School of Dentistry, University of Texas Health Science Center at Houston, 7500 Cambridge St., Houston, TX 77054, USA

**Keywords:** digital impression, intraoral scanner, mucosal thickness, removable partial denture, residual ridge, selective pressure impression

## Abstract

Although digital impression using an intraoral scanner (IOS) has been applied for removable partial denture (RPD) fabrication, it is still unclear how the morphology of a residual ridge recorded by digital impression would differ from that recorded by conventional impression. This in vivo study investigated the morphological difference in the recorded residual ridge between digital and conventional impressions. Vertical and horizontal displacements (VD and HD) in residual ridges recorded by digital and conventional impressions were assessed in 22 participants (15 female; mean age 78.2 years) based on the morphology of the tissue surface of in-use RPD. Additionally, the mucosal thickness of the residual ridge was recorded using an ultrasound diagnostic device. VD and HD were compared using the Wilcoxon signed-rank test, and the correlation of mucosal thickness with VD and HD was analyzed using Spearman’s *ρ*. The VD of digital impression was significantly greater than that of a conventional impression (*p* = 0.031), while no significant difference was found in HD (*p* = 0.322). Meanwhile, the mucosal thickness showed no significant correlation with the recorded morphology of the residual ridge, regardless of the impression techniques. It was concluded that the digital impression would result in a greater displacement in the height of the residual ridge from the morphology of in-use RPD than the conventional impression.

## 1. Introduction

Recently, the digital impression technique using an intraoral scanner (IOS) has been clinically applied for the fabrication of removable partial dentures (RPD) [1,2]. Conventionally, a selective pressure impression is recommended to fabricate well-fitted RPD with extension base(s) for Kennedy class I and II arches [3]. For conventional impressions, silicone or zinc oxide eugenol-based impression materials have been clinically used [4]. On the other hand, an IOS never places any pressure on the residual ridge while taking digital impressions because it is a non-contact system. Therefore, applying the digital impression technique to fabricate RPD for patients with Kennedy class I and II dental arches has been clinically suspicious, leading to the limitation of the application of the digital impression technique at the current moment [5]. 

In previous clinical reports with the application of digital workflow to RPD fabrication for Kennedy class I and II dental arches, clinicians have applied the conventional impression technique to obtain the definitive impression [6,7] or taken the definitive impression via an altered cast technique using an occlusion rim or a framework fabricated on the cast based on the initial digital impression [8]. In these procedures, using the combination of conventional impression techniques and digital workflow, the following benefits of digital workflow could not be obtained: reduction of physical material waste, higher cost-effectiveness, smaller number of clinical appointments, and relief of discomfort during impression taking [5]. 

To finalize the definitive impression of Kennedy class I and II dental arches using only the digital impression technique, data modification subsequent to the digital impression taking to reproduce the morphology of the residual ridge during RPD functioning would be a potential breakthrough. The conventional impression technique with selective pressure has been clinically accepted over the ages; therefore, the preference is to deal with the morphology of the residual ridge recorded using the conventional impression technique as the reference for the digital impression technique. However, no previous study has strictly compared the morphology of the recorded residual ridge between the digital and conventional impression techniques. Additionally, the mucosal thickness of the residual ridge would affect the displacement of denture-bearing mucosa under functional pressure [9], implying the importance of clarifying the influence of the mucosal thickness on the morphological displacement of the recorded residual ridge during impression taking.

In this study, the vertical and horizontal displacement in the residual ridge recorded using the digital and conventional impression techniques were assessed based on the shape of the tissue surface of in-use RPD as the measures of morphological evaluation. Additionally, the mucosal thickness of the residual ridge was assessed. This study aimed to investigate the morphological difference in the recorded residual ridge between the digital and conventional impression techniques, as well as the correlation of the mucosal thickness with the morphology of the residual ridge recorded using the digital and conventional impression techniques. The tested null hypotheses were as follows: (1) there would be no significant difference in the morphology of the residual ridge between the digital and conventional impression techniques, and (2) there would be no correlation of the mucosal thickness with the morphology of the recorded residual ridge regardless of the impression technique selection.

## 2. Materials and Methods 

### 2.1. Participants

Participants were recruited from partially edentulous patients who visited the prosthodontics clinic at the Tokyo Medical and Dental University Hospital. The inclusion criteria were as follows: mandible classified class I or class II based on the Kennedy classification of partially edentulous dental arches [10]; continuous use of mandibular RPD for at least 1 month without any complaints; and the existence of all the anterior teeth. The exclusion criteria were as follows: acute dental and periodontal disease; severe mobility of RPD abutment teeth; mucosal lesion in the residual ridge; and the existence of overdenture abutments. All participants were informed of the study purpose in written descriptions of the research protocol before they provided consent to be enrolled. All experimental procedures followed the principles of the Declaration of Helsinki, revised in 2013, and were approved by the Ethics Committee of the Tokyo Medical and Dental University (authorization no. D2020-064). The data were collected from January 2022 to September 2023.

### 2.2. Digital Impression Data

**Figure 1:**
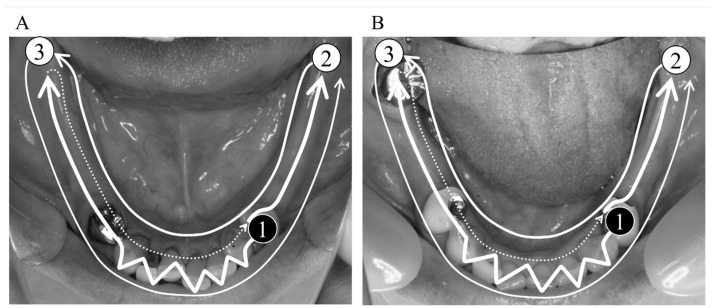
Routine process for full-arch intraoral scanning of the Kennedy class I (**A**) and class II (**B**) mandibular partially edentulous arch (assuming that the target mucosal area would be the right side). The scanning was initiated from the abutment tooth directly facing the target mucosal area (black circle numbered as #1) toward the other side with zigzag motion on the anterior teeth and returned to the target mucosal area, followed by route (#2) on the lingual surface, and then route (#3) on the buccal surface.

Each participant was instructed not to wear the mandibular RPD starting 24 h before digital impression taking so that the denture-bearing mucosa compressed by the functional pressure would be recovered. The mandible of each participant without RPD placement was digitally scanned using an IOS (TRIOS3; 3Shape, Copenhagen, Denmark) with full-arch scanning, and the acquired digital data were defined as the “digital impression data”. In accordance with a previous study, the scans were initiated from the RPD abutment tooth directly facing the target mucosal area toward the other side over the occlusal surfaces of posterior teeth and mucosal area(s) with straight motion and the incisal edges of anterior teeth with zigzag motion and returned to the target mucosal area, followed by the lingual and then the buccal surfaces (Figure 1) [11]. After acquiring the digital impression data, another intraoral scanning was performed with mandibular RPD placement to acquire “RPD placement data”. Additionally, the digital data of the mandibular RPD were acquired as “RPD data” by scanning the occlusal, polished, and tissue surfaces of the RPD using an IOS. All the acquired data were converted to standard triangulated language (STL) files. All scans were performed by a single experienced operator (E.-Y.K.).

### 2.3. Conventional Impression Data

For each participant, a custom tray was constructed on a diagnostic cast based on a snap impression using a stock tray (SANKIN IMPRESSION TRAY; Dentsply Sirona K.K., Tokyo, Japan) and a hydrocolloid (alginate) impression material (AROMA FINE PLUS; GC CORPORATION, Tokyo, Japan) taken when the participant previously visited. After taking the digital impression, the definitive impression was taken with a selective pressure impression technique using the custom tray and a silicone rubber impression material (EXAHIFLEX; GC CORPORATION, Tokyo, Japan). Using a high-strength dental stone (NEW FUJIROCK; GC CORPORATION, Tokyo, Japan), a master cast was fabricated from the definitive impression. To compare the morphology of the master cast with the IOS data, the STL data of each master cast were acquired as “conventional impression data” by digital scanning using a desktop scanner (E1; 3shape, Copenhagen, Denmark) with a manufacturer’s reported accuracy of 10 µm (ISO 12836) [12]. All the procedures to take the conventional impression and acquire the STL data of the master cast were performed by a single experienced operator (Y.I.).

### 2.4. Calculation of Representative Values for Morphological Comparison

**Figure 2:**
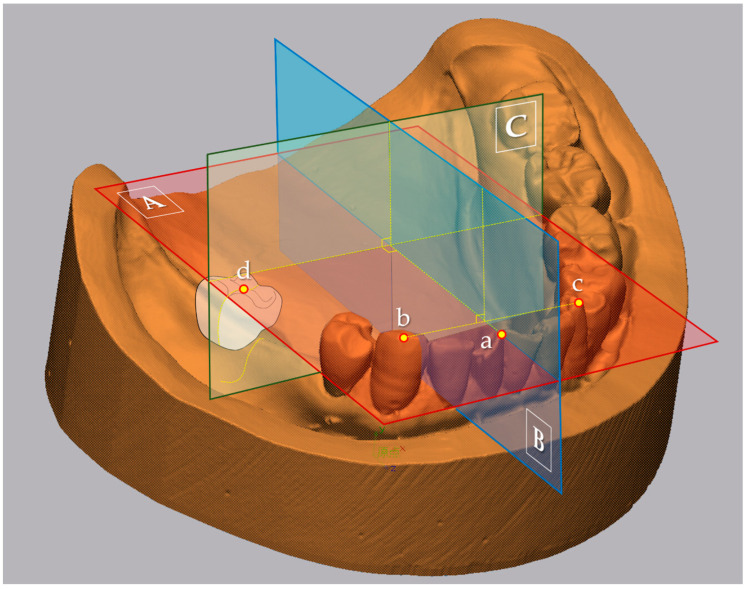
Reference and evaluation planes. (A): Reference plane #1 (RP-1) where the mesial contact point of the central incisors (a) and cusps of canines on both sides (b and c) are included; (B): Reference plane #2 (RP-2) perpendicular to the line connecting cusps of canines on both sides, and including the mesial contacting point of the central incisors; and (C): evaluation plane (EP) perpendicular to both RP-1 and RP-2, including the mesial contacting point of the central incisors, and including the central fossa of an artificial tooth of the first molar on the target mucosal area (d).

**Figure 3:**
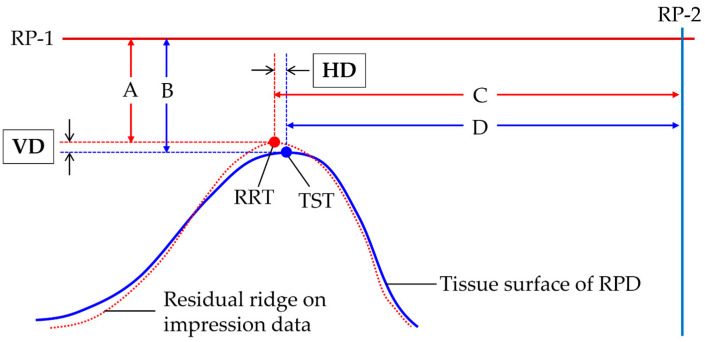
Representative values for morphological comparison. The difference in the length from RP-1 between RRT on the impression data (acquired by digital or conventional impression techniques) (A) and TST on the tissue surface of RPD (B) was defined as the vertical displacement (VD), while the difference in the length from RP-2 between RRT (C) and TST (D) was defined as the horizontal displacement (HD). RP-1: reference plane #1; PR-2: reference plane #2; RRT: residual ridge top; and TST: tissue surface top.

Using 3D analysis software (Geomagic Control X Studio 2014; 3D Systems, Rock Hill, SC, USA), the vertical and horizontal location of the residual ridge top (RRT) of the target mucosal area under the central fossa of an artificial tooth of the first molar on the RPD was evaluated. The reference planes used for the calculation of the representative values were as follows: (1) the plane including the mesial contacting point of the central incisors and cusps of canines on both sides as the reference plane #1 (RP-1) (A in Figure 2); and (2) the perpendicular plane of the line connecting cusps of canines on both sides including the mesial contacting point of the central incisors as the reference plane #2 (RP-2) (B in Figure 2). Then, the plane, including the central fossa of an artificial tooth of the first molar on the target mucosal area and perpendicular to both RP-1 and RP-2, was defined as the evaluation plane (EP) (C in Figure 2). On the EP, the highest point of the residual ridge based on RP-1 was defined as the RRT. The perpendicular line of RP-1 was drawn from RP-1 to the RRT, and the line length was recorded for both digital and conventional impressions (A in Figure 3). In addition, after superimposing the RPD data on the RPD placement data using a best-fit algorithm [13] based on the occlusal and polished surface of the RPD, the RP-1, RP-2, and the EP were defined in the same manner as for the digital and conventional impression data. Then, the highest point of the tissue surface of the RPD was defined as the tissue surface top (TST). The perpendicular line of RP-1 was drawn from RP-1 to the TST, and the line length was recorded (B in Figure 3). Finally, both for the digital and conventional impression data, the difference in the length of the perpendicular line to the VRP between RRT and TST was calculated as the vertical displacement from the tissue surface (VD). In addition to the VD, the difference in the length of the perpendicular line to the HRP between RRT (C in Figure 3) and TST (D in Figure 3) was also calculated as the horizontal displacement (HD). 

### 2.5. Mucosal Thickness of Residual Ridge

In addition to taking conventional and digital impression data, the mucosal thickness of the residual ridge (mm) was measured using an ultrasound diagnostic device (Noblus; Hitachi Medical Corporation, Tokyo, Japan) with a 13 MHz linear probe (L64 probe; Hitachi Medical Corporation, Tokyo, Japan). Each participant was instructed to sit, and the probe was placed on the assessment area so that the long axis of the probe was perpendicular to the residual ridge. The assessment area was confirmed as the area under the first molar artificial tooth based on the distance from the distal proximal surface of the abutment tooth facing the target mucosal area. The measurements were performed 5 times for each participant, and the mean value was calculated as the representative value of the mucosal thickness. All the measurements were performed by a single experienced operator (Y.I.).

### 2.6. Statistical Analysis

The VD and HD were statistically compared between digital and conventional impression data. Additionally, the correlations of the mucosal thickness with the VD and HD were statistically analyzed for the digital and conventional impression techniques. For the participants classified as Kennedy classification class I, one mucosal area was randomly selected as the target mucosal area. Levene’s test indicated that none of the measures showed the equality of data variance. Therefore, the Wilcoxon signed-rank test was performed to compare VD and HD, while Spearman’s rank correlation coefficient (*ρ*) was used to assess the correlations. All statistical analyses were performed using statistical software (SPSS Statistics v28.0, IBM, Redmond, WA, USA) with a significance level set at 0.05.

## 3. Results

### 3.1. Participants and Visual Assessment

A total of 22 participants (15 female, 7 male; mean age 78.2 ± 9.7 years) were enrolled in this study. The detailed characteristics of the participants are presented in Table 1.

### 3.2. Comparison between Digital and Conventional Impressions

**Figure 4:**
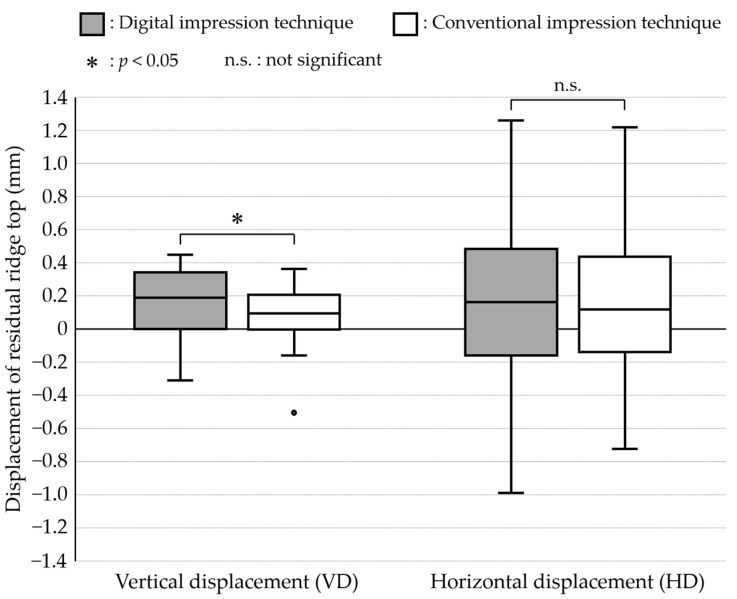
Results of comparisons of VD and HD between digital and conventional impression techniques.

Figure 4 shows the results of comparisons of VD and HD between digital and conventional impression techniques. The median values (interquartile range: IQR) (µm) of VD were 184.4 (291.1) and 93.8 (194.1) in digital and conventional impression techniques, respectively. The VD revealed significantly greater value in the digital impression data than in the conventional impression data (*p* = 0.031). On the other hand, the median values (interquartile range: IQR) (µm) of HD were 157.8 (571.2) and 115.5 (425.8) in digital and conventional impression techniques, respectively. The HD revealed no significant difference between the digital and conventional impression data (*p* = 0.322).

### 3.3. Correlation of Mucosal Thickness with the Morphological Measures

Table 2 shows the results of the correlation analyses between the mucosal thickness of the residual ridge and VD and HD with the digital and conventional impression techniques. The mucosal thickness showed no statistically significant correlation with either VD or HD with the digital and conventional impression techniques.

## 4. Discussion

In this in vivo study, the vertical and horizontal displacements (VD and HD) of the residual ridge recorded using the digital and conventional impression techniques were assessed based on the tissue surface of in-use RPD, followed by the comparisons of VD and HD between two impression techniques. Additionally, the correlations of the mucosal thickness with VD and HD were assessed for both the digital and conventional impression techniques. The overall results revealed that the digital impression technique showed a significantly greater VD than the conventional impression technique. Meanwhile, the mucosal thickness was significantly correlated only with the VD acquired with the digital impression technique. Therefore, the null hypotheses were rejected.

The vertical displacement in the mucosa of the residual ridge during distal extension RPD functioning was estimated to be around 200 μm [14]. All the RPD worn by the participants were designed based on the stress distribution concept with rigid connection of the abutment teeth and retainers without any hinges. In this RPD design concept, the denture base of the well-adjusted RPD would fit well with the compressed mucosa under functional pressure [15]. Meanwhile, the median values of the VD (the vertical gap at the RRT from the TST of in-use RPD) were 184.4 and 93.8 µm in digital and conventional impression techniques, respectively. This finding suggested that the digital impression recorded the morphology of the uncompressed mucosa, while the conventional impression using a custom tray and elastic impression material recorded the morphology of compressed mucosa to some extent. It was also suggested that the conventional impression used in this study did not provide clinically enough pressure. However, the fitness between the denture base of the in-use RPD and denture-bearing mucosa was not strictly assessed. Further studies are necessary to clarify the morphological difference between the residual ridge recorded by the digital impression and the ideal shape of the tissue surface of the distal-extension RPD defined with the exact criteria.

A previous study reported that the accuracy of static morphology of the residual ridge in the Kennedy classification class I dental model was 107–180 µm when using the digital impression [16], indicating that a potential error of more than 100 µm should be assumed regardless of the lack of pressure during impression taking. This potential error is why some participants showed negative values of VD with the digital impression technique. On the other hand, it has been indicated that the artificial marker placement could improve the accuracy of the morphology of the residual ridge recorded by the digital impression technique [17,18]. Using artificial markers would improve the potential accuracy of the digital impression technique, leading to a greater VD with the digital impression technique.

In this study, the mucosal thickness of the residual ridge was measured using an ultrasound diagnostic device with a mean value of 1.87 mm. This value was comparable to the mucosal thickness of the mandibular edentulous ridge reported in the previous study [19]. Meanwhile, the mucosal thickness showed no correlation with the vertical displacement of the RRT (VD), indicating that the digital impression technique would not be affected by mucosal thickness. It was also suggested that a lack of impression pressure in the digital impression technique would not provide clinically critical influences on the morphology of the recorded residual ridge in partially edentulous patients, indicating that distal-extension RPD fabrications made directly from the digital impression would be acceptable in those patients. On the other hand, only the participants who had used their mandibular RPD without any complaints were enrolled in this study, indicating that the denture-bearing mucosa of the participants was healthy and tightly attached to the alveolar bone. In further clinical studies, it will be necessary to compare the clinical performance of distal-extension RPD fabricated directly from the digital impressions among several allocated groups based on the mucosal thickness of the residual ridges.

Regarding the horizontal displacement of the residual ridge, a displacement of around 100 μm was found in the digital and conventional impression techniques in this study. There was no significant difference between the two impression techniques. The greater IQR found in each impression technique, as well as the similar median values, would presumably result in a lack of significant difference in the horizontal displacement. Additionally, the mucosal thickness showed no correlation with any of the evaluated measures of the horizontal displacement of the residual ridge. These findings indicated that the manual adjustment on the chairside would be needed in the horizontal morphology of the tissue surface of distal-extension RPD regardless of the selection of the impression techniques and the mucosal thickness of the residual ridge. In other words, it was indicated that the distal-extension RPD fabrication directly from the digital impression would not provide any clinically critical influences on the horizontal morphology of the tissue surface.

Overall, our findings suggested that a digital impression would provide more than a 100 µm gap in height between the tissue surface of the RPD and functionally compressed mucosa of the residual ridge, which would be around two times greater than that provided by a conventional impression. Therefore, at the moment, the digital impression technique would not be recommended for taking definitive impressions of RPD for Kennedy class I and II dental arches. On the other hand, to fabricate the RPD with full-digital procedures, modification of impression data acquired by digital impression based on the estimated gap would be a breakthrough as well as relining after RPD delivery.

This study has several limitations. First, the morphology of the residual ridge instead of the tissue surface of the fabricated RPD was evaluated. Therefore, it is still unclear whether the selection of impression techniques would critically affect the condition of the tissue surface and the clinical performance of the RPD. Second, the target area was limited to the area under the artificial tooth of the first molar as the representative area of the supporting soft tissue. The denture-bearing mucosa can be generally classified into the following two tissues: supporting tissue, which should be functionally compressed, e.g., the mucosa of the RRT, and non-supporting tissue, which requires relief, e.g., the retromolar pad. The findings of this study represent only the supporting soft tissue. Therefore, the influence of applying the digital impression technique on the non-supporting tissue could not be assessed. Although the digital impression technique might be clinically efficient in acquiring the morphology of the non-supporting tissue without any compression because of a lack of impression pressure, it is necessary to clarify how the digital impression technique affects each part of the denture-bearing mucosa, especially in Kennedy class I and II partially edentulous dental arches. Finally, this study never focused on the border area of the denture base. Conventionally, functional impressions and/or border molding with custom trays are needed to acquire the functional outline of the denture base. Further studies are necessary to assess the condition of the border area recorded using digital impression techniques and to develop a method of acquiring the proper outline of the denture base.

## 5. Conclusions

Within the limitations of this invivo study, it is suggested that the digital impression technique would result in a greater displacement in the residual ridge height from the tissue surface of in-use RPD when compared to the conventional impression technique, while the selection of the impression techniques would not affect the horizontal displacement of the residual ridge during impression taking. On the other hand, it was suggested that the morphological displacement during impression taking would not correlate with the mucosal thickness regardless of the impression techniques.

## Figures and Tables

**Table 1:** Characteristics of participants (n = 22).

Characteristic			
Age (SD)			78.2 (9.7)
Participant number (%)	Sex	Female	15 (68.2%)
		Male	7 (31.8%)
Missing teeth number of target mucosal area (%)		2	4 (18.2%)
		3	8 (36.4%)
		4	10 (45.4%)
Mucosal thickness (mm) (SD)			1.87 (0.30)

SD: standard deviation.

**Table 2:** Correlations of the mucosal thickness with the VD and HD.

Displacement	Impression Technique	*ρ*	*p*-Value
VD	Digital	−0.332	0.132
	Conventional	−0.327	0.138
HD	Digital	−0.100	0.659
	Conventional	−0.036	0.873

*ρ*: Spearman’s *ρ* with mucosal thickness of residual ridge; VD and HD are the vertical and horizontal displacements of recorded residual ridge top based on the tissue surface top of RPD, respectively.

## Data Availability

The data presented in this study are available on reasonable request from the corresponding author.
